# Case review: adult epithelial type Wilms tumor in a 23-year-old female

**DOI:** 10.3389/fonc.2026.1658242

**Published:** 2026-05-13

**Authors:** Wenchao Cui, Yan Wang, Fantao Zhang

**Affiliations:** 1 Department of Ultrasonography, Shengli Oil Field Center Hospital, Dongying, Shandong, China; 2 Mecical Imaging Department, Shengli Oil Field Center Hospital, Dongying, Shandong, China

**Keywords:** adult epithelial type, case review, early diagnosis, surgical intervention, Wilms tumor

## Abstract

**Background:**

Wilms tumor (WT) is a rare renal malignancy most commonly diagnosed in children, but it is exceedingly rare in adults. Adult Wilms tumor (AWT) presents a diagnostic challenge due to its overlap with other renal neoplasms, such as renal cell carcinoma. Among its different histological subtypes, epithelial-type Wilms tumor is even rarer, presenting with predominantly epithelial differentiation, which can mimic other epithelial renal malignancies.

**Case report:**

This case report discusses a 23-year-old female who presented with a two-year history of painless hematuria and a palpable right kidney mass. Imaging revealed a large renal mass, and the patient underwent a right radical nephrectomy. Histopathological examination confirmed the diagnosis of epithelial-type Wilms tumor, with no evidence of metastasis and clear surgical margins. Despite the rarity of this condition, the case highlights the importance of accurate histopathological diagnosis and tailored treatment strategies for adult patients with rare renal tumors. The management of adult Wilms tumors remains a subject of ongoing research, and the optimal treatment approach often requires individualized decision-making.

**Conclusion:**

Early diagnosis and surgical intervention remain critical for improving patient outcomes, and awareness of such rare tumors is essential in the differential diagnosis of renal masses in young adults.

## Introduction

Wilms tumor (WT), also known as nephroblastoma, is the most common renal malignancy in children, typically presenting before the age of 5 (1). It is a highly malignant tumor that originates from immature renal cells and is characterized by its unique histopathological features, including a mixture of blastemal, epithelial, and stromal elements (1, 2). Although Wilms tumor is almost always confined to the kidney, rare cases of primary extrarenal Wilms tumor have been reported, most commonly in pediatric patients 3. While the majority of Wilms tumors occur in childhood, their presentation in adults is extremely rare, accounting for less than 5% of all Wilms tumor cases (3). In adult patients, WT often presents as a diagnostic challenge due to its overlapping clinical and radiological features with other more common adult renal cancers, such as renal cell carcinoma (RCC) and clear cell sarcoma of the kidney (CCSK) (3, 4).

The adult form of Wilms tumor differs from its pediatric counterpart in several key ways. While pediatric Wilms tumors often have a favorable prognosis with surgical intervention and chemotherapy, adult Wilms tumors tend to present at a more advanced stage, and their prognosis is less predictable (5–8). Histologically, adult Wilms tumors may show predominant epithelial differentiation or a mixture of epithelial and stromal elements, which can complicate the diagnosis and differentiation from other renal neoplasms (9). Furthermore, the rarity of adult Wilms tumor means that treatment regimens have not been as thoroughly studied, and much of the management relies on case reports and expert opinions rather than large clinical trials.

Epithelial-type Wilms tumor is an even rarer variant of this disease. In adult patients, the epithelial predominance in the tumor is often misinterpreted as renal cell carcinoma or other epithelial renal malignancies, leading to diagnostic delays (10, 11). This highlights the importance of careful histopathological examination, as well as the need for awareness of such rare conditions among clinicians. This case report aims to highlight the clinical features, surgical management, and pathological findings of epithelial-type Wilms tumor in an adult patient, emphasizing the importance of early detection, accurate diagnosis, and tailored therapeutic approaches in managing this uncommon disease.

## Case presentation

A 23-year-old previously healthy Chinese female presented to our hospital with a chief complaint of intermittent painless gross hematuria for two years, which had progressively worsened over the past month with increased frequency and volume of blood in the urine. She also reported noticing a gradually enlarging, nontender palpable mass in the right flank over the same period. There were no associated symptoms such as flank pain, fever, weight loss, fatigue, dysuria, urinary frequency/urgency, or constitutional symptoms. She denied any recent trauma, urinary tract infections, or episodes of renal colic.

Past medical history was unremarkable, with no prior surgeries, chronic illnesses, hypertension, diabetes, or known renal disease. She had no history of radiation exposure, chemotherapy, or immunosuppressive therapy. Family history was negative for renal malignancies, hereditary cancer syndromes (e.g., WAGR, Beckwith-Wiedemann), or other relevant neoplasms. Social history included no tobacco, alcohol, or illicit drug use; she was a nonsmoker and worked as an office employee with no occupational exposures.

On physical examination, the patient was hemodynamically stable with normal vital signs. Abdominal inspection revealed mild asymmetry with a visible bulge in the right flank. Palpation confirmed a large, firm, nontender, ballotable mass in the right upper quadrant and flank, approximately 12–15 cm in size, with no overlying skin changes or bruit. No peripheral edema, lymphadenopathy, or signs of inferior vena cava obstruction were noted. The remainder of the systemic examination was unremarkable.

Laboratory evaluation included urinalysis showing significant microscopic hematuria (red blood cells +++), proteinuria (protein +++), and hemoglobinuria (hemoglobin +++), with no leukocytes or nitrites suggestive of infection. Complete blood count, renal function tests (serum creatinine and blood urea nitrogen), liver function tests, and coagulation profile were within normal limits.

Laboratory evaluation revealed significant hematuria, proteinuria, and hemoglobinuria on urinalysis, consistent with renal parenchymal involvement. Additional blood tests, including complete blood count, basic metabolic panel, inflammatory markers, and selected tumor markers, were performed as part of the routine work-up for a suspected renal malignancy. Results are summarized in **Table 1**. No abnormalities suggestive of infection, systemic inflammation, or other neoplasms were identified, and renal function remained preserved despite hydronephrosis.

**Table 1:** Laboratory findings at presentation.

Parameter	Result	Reference range (adult)	Notes
Complete Blood Count (CBC)			
Hemoglobin (g/dL)	12.5	12.0–15.5 (female)	Mild normocytic anemia possible from chronic hematuria
White blood cell count (×10^9^/L)	6.8	4.0–11.0	Normal
Platelet count (×10^9^/L)	280	150–450	Normal
Inflammatory Parameters			
C-reactive protein (CRP, mg/L)	<5.0	<5.0	No significant inflammation
Erythrocyte sedimentation rate (ESR, mm/h)	12	0–20 (female)	Normal
Renal Function & Electrolytes			
Serum creatinine (μmol/L)	68	44–80 (female)	Normal
Blood urea nitrogen (BUN, mmol/L)	4.2	2.5–7.1	Normal
Neoplastic Markers			
Alpha-fetoprotein (AFP, ng/mL)	2.1	<8.0	Normal (to exclude germ cell tumor)
Beta-human chorionic gonadotropin (β-hCG, mIU/mL)	<1.0	<5.0	Normal
Lactate dehydrogenase (LDH, U/L)	220	140–280	Normal (LDH may be elevated in some WT cases but not diagnostic)
Other			
Coagulation profile (PT/INR, aPTT)	Normal	–	No coagulopathy
Immunological findings (e.g., ANA, if tested)	Not performed	–	No indication for autoimmune work-up

All values were within normal limits except as noted. No specific serum tumor markers or immunological abnormalities are characteristic of Wilms tumor; these tests were performed to exclude differentials (e.g., infection, germ cell tumor, or paraneoplastic syndromes).

**Figure 1:**
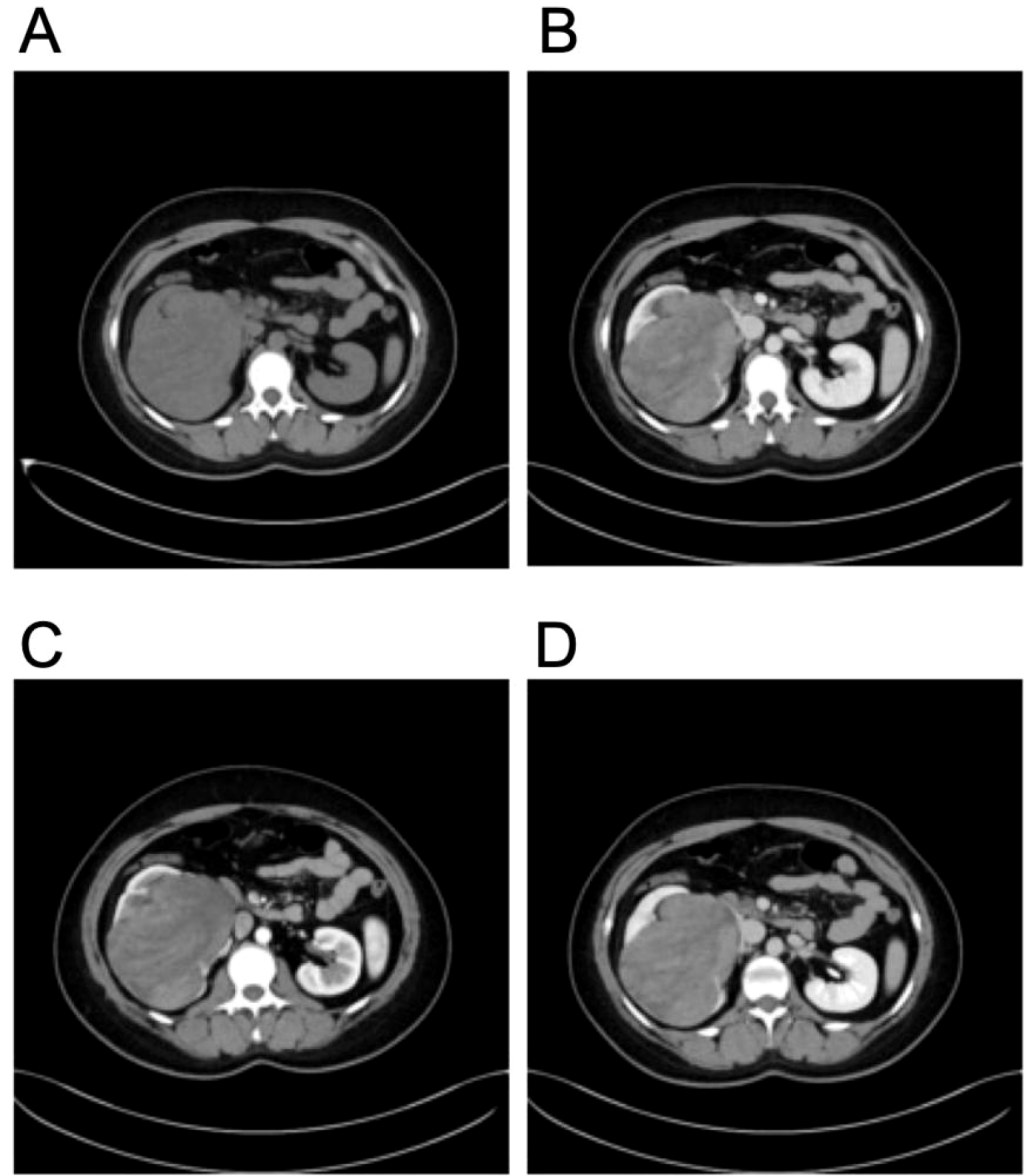
Representative contrast-enhanced computed tomography images of the large right renal mass in a 23-year-old female. **(A)** Axial non-contrast phase: A heterogeneous soft tissue mass measuring approximately 11.2 × 8.4 cm occupies a substantial portion of the right kidney, with no evidence of calcification, liquefaction, or obvious necrosis. **(B)** Axial corticomedullary/arterial phase: The mass demonstrates moderate and uneven enhancement, most prominent in the peripheral and septated-like internal components, with relative hypoattenuation in central areas. **(C)** Axial venous/portal phase: Persistent heterogeneous enhancement is seen throughout the mass, with continued uneven distribution and no washout pattern suggestive of simple cyst. **(D)** Coronal reformatted view: The mass causes significant mass effect on the kidney, with compression and displacement of the renal parenchyma, particularly evident in the lower pole and medial aspect, and extension toward the renal hilum/pelvis.

**Figure 2:**
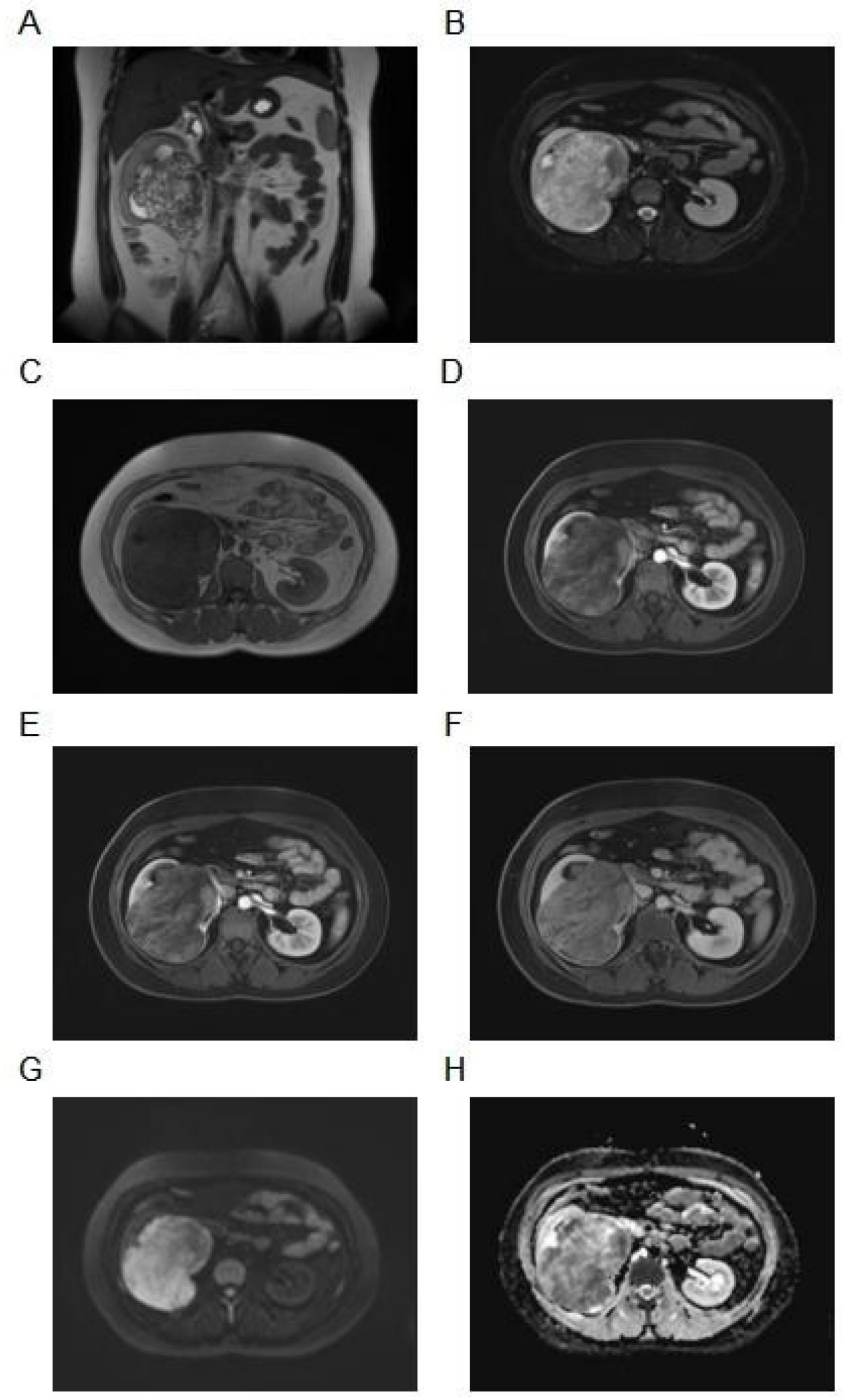
MRI analysis images. **(A)** Coronal T2-weighted imaging (T2WI): Abnormal high signal intensity within the mass, accompanied by marked dilation of the right renal pelvis (hydronephrosis) and thinning of the overlying renal parenchyma. **(B)** Axial fat-saturated T2WI: Mixed high and intermediate signal in the mass, with clear compression and thinning of the surrounding renal parenchyma, most evident laterally and superiorly. **(C)** Axial T1-weighted imaging (T1WI): Predominantly low-to-intermediate signal intensity throughout the lesion. **(D)** Contrast-enhanced arterial phase: Heterogeneous and uneven enhancement, more pronounced in peripheral zones. **(E)** Venous phase: Persistent uneven enhancement pattern. **(F)** Delayed phase: Delayed enhancement of the mass component extending into the proximal ureter, with associated upstream hydronephrosis. **(G)** Diffusion-weighted imaging (DWI, b=800): High signal intensity in most of the lesion, consistent with restricted diffusion. **(H)** Apparent diffusion coefficient (ADC) map: Corresponding low ADC values confirming restricted diffusion in the bulk of the mass.

**Figure 3:**
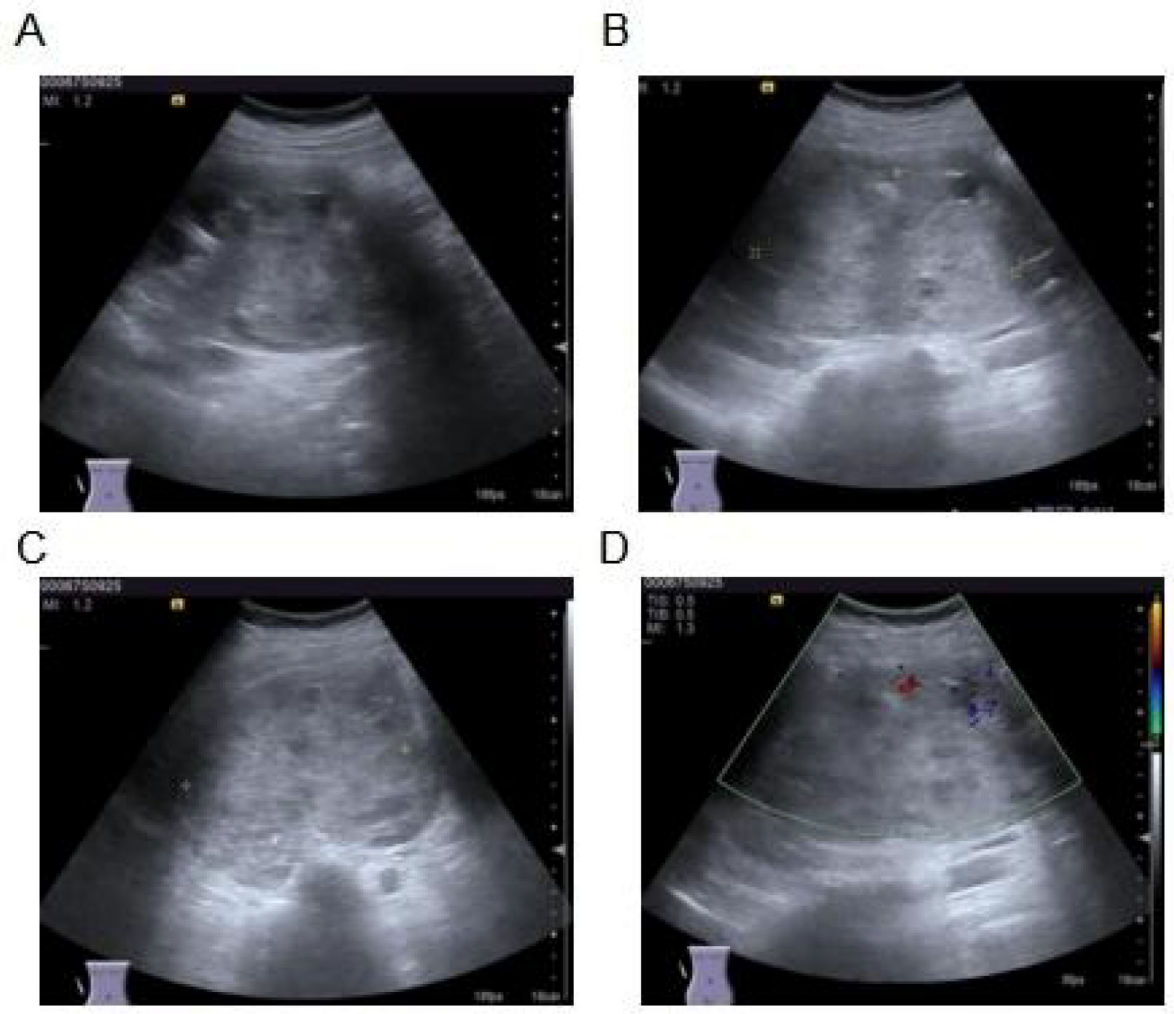
Ultrasound analysis images. **(A)** Longitudinal view: A large isoechoic mass (measuring approximately 120 × 104 × 75 mm) centered in the renal pelvis region, distorting the normal renal architecture. **(B)** Long-axis view: Uneven internal echo distribution with scattered small cystic necrotic areas within the mass. **(C)** Short-axis/transverse view: The mass has relatively clear boundaries and causes marked compression and thinning of the adjacent renal parenchyma. **(D)** Color Doppler flow imaging (CDFI): Minimal to no detectable internal vascularity within the mass.

Imaging studies were performed for further evaluation. Contrast-enhanced computed tomography (CT) of the abdomen revealed a heterogeneous soft tissue mass in the right kidney measuring approximately 11.2 × 8.4 cm, with moderate and uneven enhancement but no liquefaction, necrosis, or calcification (**Figure 1**). Magnetic resonance imaging (MRI) demonstrated mixed T1 and abnormal T2 signals, with high signal on diffusion-weighted imaging (DWI, b=800) and restricted apparent diffusion coefficient (ADC) values in most of the lesion. The mass extended into the ureter with delayed enhancement, causing compression and thinning of the renal parenchyma, dilation of the right renal pelvis, and hydronephrosis (**Figure 2**). Abdominal ultrasound showed an enlarged, abnormally shaped right kidney with a large isoechoic mass (120 × 104 × 75 mm) centered in the renal pelvis region, uneven internal echoes, small cystic necrotic areas, relatively clear boundaries, and minimal internal vascularity on color Doppler flow imaging (CDFI). The surrounding parenchyma was compressed and thinned (**Figure 3**). Preoperative staging followed adapted pediatric Wilms tumor protocols to exclude metastatic disease and assess resectability. This included contrast-enhanced abdominal CT/MRI (**Figures 1**, **2**) to evaluate local extension, vascular involvement (renal vein/IVC), contralateral kidney, and liver metastases; dedicated chest CT to rule out pulmonary metastases (most common site); and routine blood work (**Table 1**). No distant metastases or extensive vascular thrombus (e.g., suprahepatic IVC or atrial extension) were identified, confirming suitability for upfront surgical resection.

**Figure 4:**
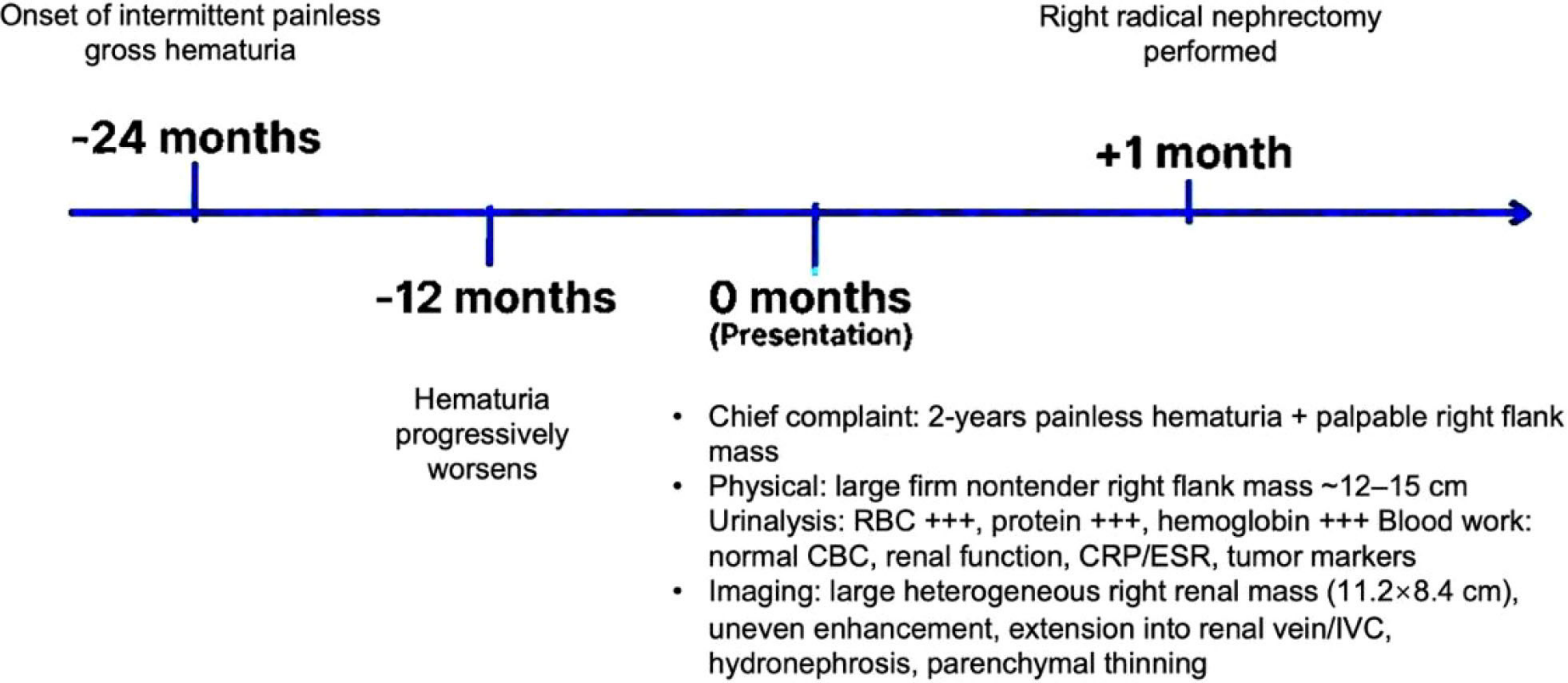
Timeline summarizing the longitudinal clinical course of the patient, highlighting key events: onset and progression of hematuria, presentation with clinical/laboratory/radiological findings, and surgical intervention with pathologic outcome.

Based on the clinical presentation, laboratory findings, and multimodality imaging suggestive of a malignant renal neoplasm (differential including renal cell carcinoma, Wilms tumor, or sarcoma), the multidisciplinary team recommended surgical intervention. The overall progression from symptom onset to definitive management is illustrated in **Figure 4**.

## Surgical procedure

Given the clinical findings and imaging results, the patient underwent a right radical nephrectomy. The surgery was performed under general anesthesia with the patient in a supine position. A laparoscopic approach was used to establish pneumoperitoneum and facilitate the removal of the right kidney. Careful dissection was performed to preserve critical structures, such as the ureter and surrounding vasculature, and the kidney was excised along with part of the ureter and a segment of the bladder wall involved with the lesion.

The surgery was completed without complications, and the specimen was sent for histopathological examination. The postoperative course was uneventful, with no significant bleeding or infection observed.

## Histopathological examination

**Figure 5:**
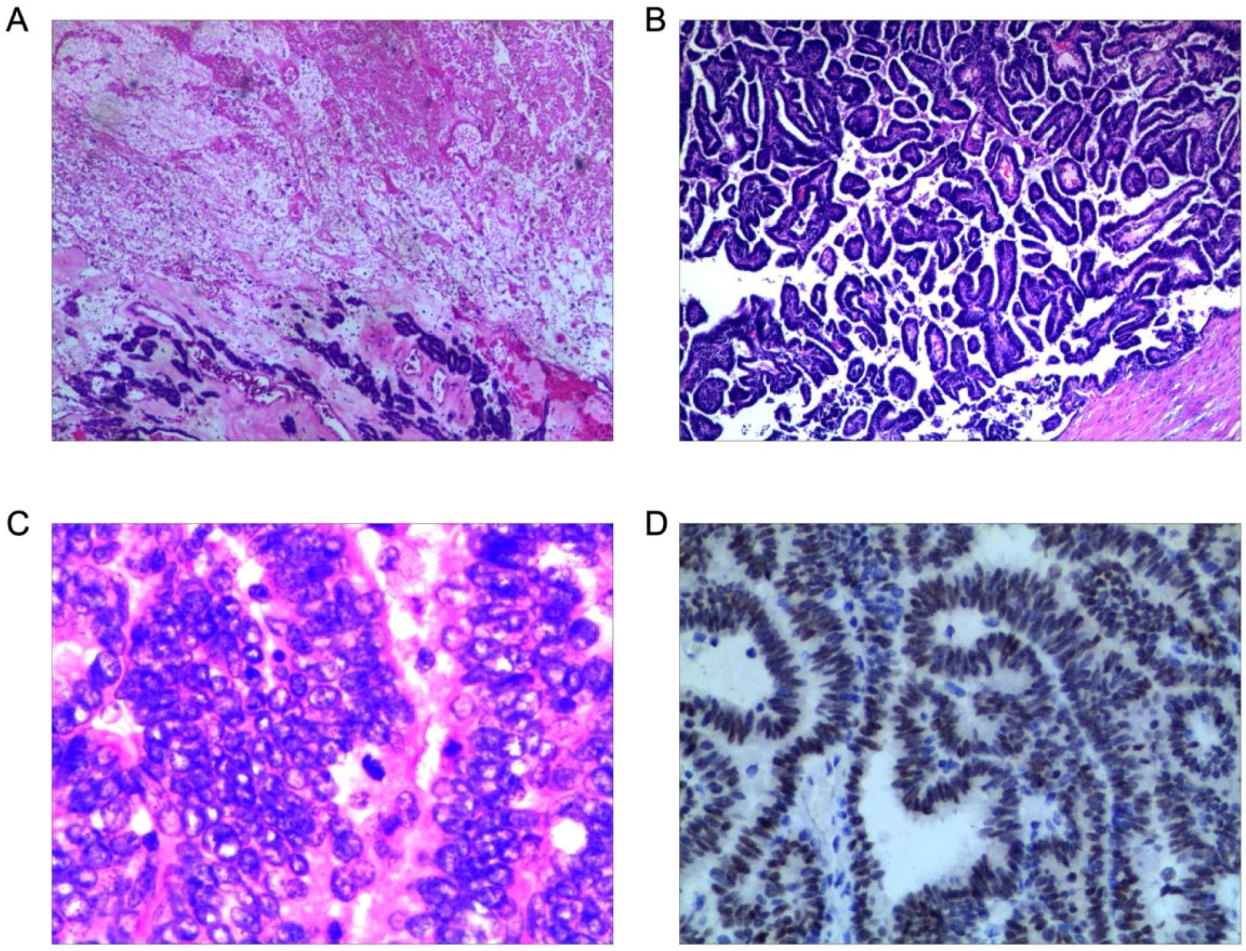
Histopathological and immunohistochemical findings of the epithelial-type Wilms tumor. **(A)** Low-power view (×40) demonstrating areas of tumor necrosis. **(B)** Low-power view (×40) showing epithelial-like tumor cells with papillary growth pattern. **(C)** High-power view (×400) revealing tumor cells with eosinophilic cytoplasm, vacuolated nuclei, and mitotic figures. **(D)** Immunohistochemistry (EliVision method) showing strong nuclear positivity for WT-1 in tumor cells.

The pathological examination of the excised kidney confirmed epithelial-type Wilms tumor. The tumor exhibited predominantly epithelial differentiation with tubular, papillary, and glomerular structures. At low magnification (×40), areas of tumor necrosis were evident (**Figure 5A**), the tumor cells displayed a papillary growth pattern (**Figure 5B**). High-power examination (×400) revealed tumor cells with eosinophilic cytoplasm, vacuolated nuclei, moderate nuclear pleomorphism, prominent nucleoli, and identifiable mitotic activity (**Figure 5C**). Immunohistochemistry demonstrated strong nuclear positivity for WT-1 in tumor cells (**Figure 5D**).The tumor was confined to the kidney with no regional metastasis, and resection margins were negative. A pseudocapsule was present, with focal compression of adjacent renal parenchyma and extension into the ureter, indicating invasive behavior.

The tumor was confined to the kidney, with a pseudocapsule present and focal compression of adjacent renal parenchyma. There was no invasion of the renal sinus soft tissues or renal sinus vessels/lymphatics. No lymphovascular invasion was identified within the renal parenchyma or hilar structures. No regional lymph node involvement was present in the submitted specimen. Resection margins (including vascular, ureteral, and soft tissue) were negative for malignancy. These features are consistent with pathologic Stage I disease (per COG/NWTS staging criteria for Wilms tumor, adapted for adult presentation: tumor limited to the kidney without capsular penetration, renal sinus involvement, or lymphovascular invasion beyond intrarenal vessels; complete resection achieved). Regional hilar and perirenal lymph nodes were sampled intraoperatively as recommended in adapted COG/NWTS protocols for Wilms tumor. A total of 4 lymph nodes were submitted and examined; all were negative for metastatic disease, supporting accurate pathologic staging.

To exclude metanephric adenoma—a major benign differential diagnosis for epithelial-predominant renal tumors in adults, particularly given overlapping WT-1 positivity—the following features were decisive: metanephric adenoma typically shows cytologically bland, uniform small cells with scant mitoses, absent or minimal atypia, rare necrosis, and often psammoma bodies with a non-infiltrative border. In contrast, this case demonstrated brisk mitotic activity, nuclear atypia/vacuolization, tumor necrosis, papillary architecture with pleomorphism, and invasive ureteral involvement—features favoring malignancy and consistent with epithelial-predominant Wilms tumor rather than metanephric adenoma.

Adult Wilms tumors remain challenging to distinguish from other renal neoplasms (e.g., clear cell sarcoma, renal cell carcinoma) due to histologic overlap, but the combination of epithelial predominance, mitotic activity, necrosis, and WT-1 positivity supported the diagnosis in this case.

## Discussion

Adult Wilms tumor (AWT) remains an exceedingly rare entity, representing <5% of all Wilms tumor cases and <0.2 cases per million adults annually. Epithelial-predominant variants are even scarcer in adults, frequently mimicking more common renal cell carcinoma (RCC) or metanephric adenoma due to predominant tubular/papillary architecture, WT-1 positivity, and lack of prominent blastemal/stromal elements. This diagnostic overlap often leads to misdiagnosis or delayed recognition, contributing to advanced presentation and poorer outcomes compared to pediatric Wilms tumor.

The present case of a 23-year-old female with a 2-year history of intermittent painless gross hematuria, culminating in a palpable flank mass and large right renal lesion with ureteral extension and hydronephrosis, exemplifies these challenges. Notably, the prolonged symptom duration without pain, constitutional symptoms, or metastatic spread at diagnosis is atypical for AWT, which more commonly presents at advanced stages. Multimodality imaging (heterogeneous enhancement on CT, restricted diffusion and ureteral involvement on MRI, isoechoic pelvic mass with cystic necrosis and minimal vascularity on US) raised suspicion for malignancy but was nonspecific for histologic subtype. Only careful histopathological evaluation—revealing papillary epithelial growth, eosinophilic cytoplasm with vacuolated nuclei, brisk mitoses, tumor necrosis, and strong nuclear WT-1 positivity—confirmed epithelial-type Wilms tumor. Critically, the presence of mitotic activity, atypia, necrosis, and invasive behavior (ureteral extension) excluded metanephric adenoma, a key benign mimic in adults that typically shows bland cytology, absent mitoses, and often psammoma bodies or BRAF V600E mutation.

Clear cell sarcoma of the kidney (CCSK), another important differential, was excluded based on the absence of classic histologic features such as cord-like patterns, chicken-wire vasculature, and osteoid-like matrix (12). Historical pathologic reviews further support the distinct separation of epithelial-predominant Wilms tumor from related childhood renal neoplasms, including mesoblastic nephroma variants occasionally seen in adults (13, 14).

This case underscores several practical implications for clinical practice. First, in young adults with renal masses and prolonged painless hematuria, AWT—though rare—should enter the differential alongside RCC, especially when imaging shows heterogeneous enhancement without classic RCC features (e.g., no washout). Second, upfront radical nephrectomy (as performed here) achieved complete resection with negative margins and no metastasis, supporting pathologic Stage I classification (no renal sinus invasion, no lymphovascular involvement) per adapted COG/NWTS criteria used in pediatric and young adult populations (15, 16). This favorable pathologic stage, combined with epithelial predominance, aligns with observations that such variants may confer relatively better outcomes in select adult cases compared to blastemal-rich or advanced AWT. Third, the absence of adjuvant chemotherapy in this Stage I case highlights the individualized approach necessitated by limited adult-specific data; pediatric protocols are often extrapolated, but decisions require multidisciplinary input weighing risks/benefits (17, 18).

Preoperative staging in adult Wilms tumor, though not standardized, adapts pediatric COG/NWTS recommendations emphasizing exclusion of metastases (lungs via chest CT, liver via abdominal imaging) and assessment of vascular extension (renal vein/IVC degree via CT/MRI) before surgery. Mandatory imaging includes contrast-enhanced abdominal CT/MRI and chest CT; extensive IVC/atrial involvement may warrant neoadjuvant therapy or specialized vascular surgery. In our case, negative metastatic workup and limited vascular involvement supported immediate radical nephroureterectomy, achieving favorable Stage I disease. The treatment of adult Wilms tumor lacks dedicated guidelines and adapts pediatric protocols. The NWTS/COG approach favors upfront nephrectomy for resectable localized disease, enabling precise pathologic staging and histologic confirmation—particularly valuable in adults where differentials include RCC and metanephric adenoma. In contrast, the SIOP-RTSG 2016 protocol recommends preoperative chemotherapy (vincristine-actinomycin D) for most cases to reduce tumor volume and spill risk, followed by risk-adapted adjuvant therapy. In adults, upfront surgery is more commonly reported due to diagnostic challenges and uncertain chemotherapy response in non-pediatric biology. Our case followed the COG-adapted strategy: preoperative imaging excluded metastases and extensive vascular extension, allowing immediate radical nephroureterectomy with lymph node sampling, achieving Stage I disease without adjuvant treatment.

The novelty of this report lies in the documentation of epithelial-predominant AWT presenting with a remarkably indolent 2-year symptomatic course in a very young adult, successful exclusion of metanephric adenoma through morphologic criteria (mitoses, necrosis, atypia) despite shared WT-1 expression, and achievement of Stage I disease with surgery alone. While large trials are lacking, such cases reinforce the importance of heightened awareness, precise histopathological assessment (including IHC panels when overlapping features exist), and tailored management to optimize outcomes in this uncommon malignancy (19). To contextualize this case within the sparse literature on epithelial-predominant adult Wilms tumor, we reviewed recent reports (2015–2025). **Table 2** summarizes key published adult cases of epithelial-predominant or epithelial-type WT, focusing on demographics, presentation, diagnostic challenges, treatment, and outcomes (10, 20–22).

**Table 2:** Summary of reported adult cases of epithelial-predominant/epithelial-type Wilms tumor.

Year/reference	Age/sex	Presentation/duration	Key histology/IHC	Stage/size	Treatment	Outcome/follow-up	Notable features/differential notes
2013 (Watanabe et al., ref (20)	Adult (3 cases)	Renal mass (details limited)	Predominant epithelial component; papillary/tubular	Not specified	Nephrectomy (assumed)	Not detailed	Differential: papillary RCC, metanephric adenoma
2024 (Chapman et al., ref (10)	30/M	Large renal mass, elevated creatinine	Epithelial-predominant WT; WT-1+	Not specified/11.3 × 7.5 × 12.6 cm	Radical nephrectomy	Not detailed	Associated with Jeune syndrome; discusses favorable prognosis in epithelial type
2025 (He et al., ref (21)	Adult (2 cases in series)	Renal mass (details limited)	Epithelial type; necrosis in one case	Not specified	Nephrectomy ± other	5-year follow-up available	Part of 4-case adult WT series; necrosis but no perirenal invasion
2025 (Kolap et al., ref (22)	Adult	Renal mass, biphasic features	Biphasic (epithelial + blastemal)	Not specified	Nephrectomy	Not detailed	Mentions epithelial component and BRAF overlap in some adult WT
Present case	23/F	2-year intermittent painless gross hematuria + palpable flank mass	Epithelial-predominant; papillary growth, mitoses, necrosis, WT-1+	Stage I/~11.2 × 8.4 cm	Right radical nephrectomy alone	No complications, no metastasis	Indolent 2-year course, young age, morphologic exclusion of metanephric adenoma

This report describes an unusually indolent presentation of epithelial-predominant adult Wilms tumor in a young adult, characterized by a 2-year history of painless hematuria without systemic symptoms or metastasis, ultimately diagnosed as Stage I disease after radical nephrectomy with clear margins. The case highlights the critical role of meticulous histopathological evaluation—including recognition of mitotic activity, necrosis, and atypia—in distinguishing epithelial-type Wilms tumor from benign mimics such as metanephric adenoma, even when WT-1 immunohistochemistry is shared. Clinicians should maintain awareness of adult Wilms tumor in the differential diagnosis of renal masses in young adults with prolonged hematuria, as early surgical intervention can achieve favorable outcomes in low-stage epithelial-predominant cases despite the limited evidence base for adjuvant therapy in adults.
